# A risk-based model to assess environmental justice and coronary heart disease burden from traffic-related air pollutants

**DOI:** 10.1186/s12940-020-00584-z

**Published:** 2020-03-16

**Authors:** James P. Fabisiak, Erica M. Jackson, LuAnn L. Brink, Albert A. Presto

**Affiliations:** 1 Center for Healthy Environments & Communities, Department of Environmental & Occupational Health, University of Pittsburgh Graduate School of Public Health, PUBHL-4132, 130 DeSoto Street Pittsburgh, Pittsburgh, PA 15261 USA; 2 Allegheny County Health Department, Pittsburgh, PA USA; 3 Center for Atmospheric Particle Studies, Department of Mechanical Engineering, Carnegie Mellon University, Pittsburgh, PA USA

**Keywords:** Environmental justice, Air pollution, Nitrogen dioxide, Black carbon, Coronary heart disease, Risk assessment, Land use regression modeling

## Abstract

**Background:**

Communities need to efficiently estimate the burden from specific pollutants and identify those most at risk to make timely informed policy decisions. We developed a risk-based model to estimate the burden of black carbon (BC) and nitrogen dioxide (NO_2_) on coronary heart disease (CHD) across environmental justice (EJ) and non-EJ populations in Allegheny County, PA.

**Methods:**

Exposure estimates in census tracts were modeled via land use regression and analyzed in relation to US Census data. Tracts were ranked into quartiles of exposure (Q1-Q4). A risk-based model for estimating the CHD burden attributed to BC and NO_2_ was developed using county health statistics, census tract level exposure estimates, and quantitative effect estimates available in the literature.

**Results:**

For both pollutants, the relative occurrence of EJ tracts (> 20% poverty and/or > 30% non-white minority) in Q2 – Q4 compared to Q1 progressively increased and reached a maximum in Q4. EJ tracts were 4 to 25 times more likely to be in the highest quartile of exposure compared to the lowest quartile for BC and NO_2_, respectively. Pollutant-specific risk values (mean [95% CI]) for CHD mortality were higher in EJ tracts (5.49 × 10^− 5^ [5.05 × 10^− 5^ – 5.92 × 10^− 5^]; 5.72 × 10^− 5^ [5.44 × 10^− 5^ – 6.01 × 10^− 5^] for BC and NO_2_, respectively) compared to non-EJ tracts (3.94 × 10^− 5^ [3.66 × 10^− 5^ – 4.23 × 10^− 5^]; 3.49 × 10^− 5^ [3.27 × 10^− 5^ – 3.70 × 10^− 5^] for BC and NO_2_, respectively). While EJ tracts represented 28% of the county population, they accounted for about 40% of the CHD mortality attributed to each pollutant. EJ tracts are disproportionately skewed toward areas of high exposure and EJ residents bear a greater risk for air pollution-related disease compared to other county residents.

**Conclusions:**

We have combined a risk-based model with spatially resolved long-term exposure estimates to predict CHD burden from air pollution at the census tract level. It provides quantitative estimates of effects that can be used to assess possible health disparities, track temporal changes, and inform timely local community policy decisions. Such an approach can be further expanded to include other pollutants and adverse health endpoints.

## Background

The adverse health effects from elevations in ambient air pollution are well established. Notable pollutants of concern, for which National Ambient Air Quality Standards are set by the US EPA, include ozone, sulfur dioxide, nitrogen dioxide (NO_2_), lead, carbon monoxide and particulate matter (PM). Separate standards for PM exist based on its size profile; PM_10_ (< 10 μm mean aerodynamic diameter, MADD) and PM_2.5_ (< 2.5 μm MADD), with smaller particles being the primary health concern. In contrast to the other pollutants, PM is chemically and physically diverse depending in large part on its source.

The relationship between exposure to air pollution and adverse health outcomes, some severe, has been demonstrated now in multiple epidemiological and other studies. Elevations in exposure to several of the criteria air pollutants have been associated with premature overall mortality [1, 2], exacerbation of respiratory disease like asthma [3–5], adverse birth outcomes [6, 7], increased rate of hospitalizations [8, 9] and death from cardiovascular disease [10–12]. Practical limitations in routinely conducting these large-scale health effect studies, however, include the requirements of a large population and long study duration in order to achieve statistical power, sufficient access to patient health records over the study period, and exposure estimates are often limited by poor spatial resolution.

Pittsburgh, a major US city, sits in Allegheny County located in the southwest corner of Pennsylvania. The region has a legacy of heavy industrialization, and even today, air pollution remains problematic compared to many other areas of the US [13–15]. Like any urban center, residents are exposed to pollutants such as diesel exhaust particulates and NO_2_ from the high density of motor vehicles. Uniquely, Allegheny County still contains numerous high-emitting industrial point sources of pollution mostly related to the coal, steel, and metals industries. In addition, long-range transport from other industrial centers in the Midwest and a propensity for meteorological thermal inversions in low-lying river valley areas trapping pollutants also add to the burden. Parts of Allegheny County remain in non-attainment for PM_2.5_ and, along with some surrounding counties, are in non-compliance of the SO_2_ standard [16]. Removal of ozone non-attainment status for Southwest PA has only recently been recommended by the PA Dept. of Environmental Protection and is pending EPA approval [17]. It is also important to consider that with limited spatial distribution of regulatory air monitors and, the fact that recent large population studies [18, 19] indicate health effects are appreciable at levels below ambient standards, it is likely that air pollution exposures and, hence, health burden are heterogeneously distributed across the region.

Therefore, there is continuing and increasing need for communities to be able to estimate the potential impact of air pollution on their residents. Based on the technical limitations of conducting adequate large-scale epidemiological studies described above, it is often impractical to gather empirical disease incidence data to inform decisions in a timely fashion and as they may relate to neighborhood heterogeneity. Many of the epidemiological studies performed elsewhere, however, do provide quantitative effect estimates describing the relationship between exposure level and disease incidence that can then be applied in risk-based models to estimate the disease burden associated with specific exposures.

While spatial maps of pollutants have been employed in epidemiological studies that associate incidence of disease with exposure [20, 21], few studies have linked spatial gradients of exposure at predicting or modeling disease at the community and neighborhood level using low-cost, time-efficient risk-based models. Estimations of disease burden from air pollution exist but these are frequently calculated at the global level [22, 23]. Correlation of spatial maps of NO_2_ exposure with demographics have revealed disparities by race and income over the entire U.S. [24, 25], however, more granular insight and consideration of health inequities is needed for policy to advance at the community level.

We sought to develop a risk-based model that would allow us to combine existing land use regression (LUR) models describing the geographic distribution of particular pollutants within Allegheny County with high spatial resolution along with published health effect estimates [20]. From this we could estimate air pollution-dependent disease burdens at the census tract level, including those designated as environmental justice (EJ) tracts based on race and income. We selected NO_2_ and black carbon (BC) as two model pollutants. NO_2_ derives primarily from both gasoline and diesel-powered motor vehicles with smaller contributions coming from industrial sources, in particular regional coal-fired power plants [15]. BC is a component of particulate matter and serves as a surrogate for the combustion-related PM_2.5_ components [26]. Primary PM sources in Allegheny County again include traffic-related particulates primarily from diesel exhaust, but also significant contributions from industrial point sources especially related to coke processing, steel manufacture, metal fabrication, as well as, residential wood burning [14]. Both pollutants are associated with adverse cardiovascular events and we chose increased mortality and hospitalization from coronary heart disease as a specific health endpoint of interest [20, 27, 28]. By coupling spatial exposure and health effect estimates with demographic US census data we calculate the hypothetical excess CHD burden arising from NO_2_ and BC exposure and show that EJ sensitive areas are were more numerous in areas of higher exposure and bear a disproportionate amount of risk of CHD from air pollution compared to non-EJ areas.

## Methods

### Land use regression exposure estimates

Pollutant exposures were assigned based on land use regression (LUR) models for BC and NO_2_. LUR modeling and pollutant data collection are described by Tan et al. [29] and Li et al. [30]. Briefly, pollutant data was collected via mobile sampling to measure pollutants including BC (Magee Scientific AE31 Aethalometer) and NO_2_ (Teledyne API T200). Mobile sampling was conducted at 42 sites in Allegheny County during winter (2011–2012) and summer (2012) with each site sampled in the morning (5–11 AM), afternoon/evenings (11 AM – 9 PM) and overnight (9 PM – 5 AM) in each season. The sites were selected using random sampling stratified by elevation (valley or upland) and traffic volume (high or low traffic). Eight sites were valley sites with low traffic, 11 sites were valley sites with high traffic, 13 sites were upland sites with low traffic, and 10 sites were upland sites with high traffic. The mobile laboratory was driven along a prescribed driving route at each site.

The time series of BC data includes many short-duration spikes attributable to diesel vehicles [31]. Thus, we constructed, as described by Tan et al. [29], a hybrid BC LUR that separates short-term spikes from the rest of the BC signal. Spikes are attributed to on-road diesel traffic, and the remaining signal is used to construct an LUR model for the background (non-traffic) BC. The final BC spatial model is the sum of the vehicle plume layer and the background LUR.

We constructed a separate LUR for NO_2_. The NO_2_ time series did not exhibit high concentration spikes, therefore we did not decompose the signal as with BC. The LUR model was fit to the mean NO_2_ concentration at each of the 42 sampling locations.

The background BC LUR and the NO_2_ LUR both follow the stepwise variable selection process used in the ESCAPE project [32]. Each land-use covariate is assigned a prior direction, either a positive or negative regression coefficient. For example, traffic emissions contribute to NO_2_, so all covariates related to vehicle traffic are assumed to have a positive coefficient. Land-use covariates are added to the model if they increase the model R^2^ by more than 0.01. Variables are removed if their *p*-value is greater than 0.1 or if the variance inflation factor is greater than 3. Both NO_2_ and BC spatial models were evaluated using leave one out cross validation and by testing predictions against a hold-out dataset collected at an additional 30 sites in 2013–2014. The BC and NO_2_ LURs are representative of long-term (e.g., annual) average concentrations. The R^2^ for the BC model is 0.67, and 0.76 for the NO_2_ model.

**Fig. 1:**
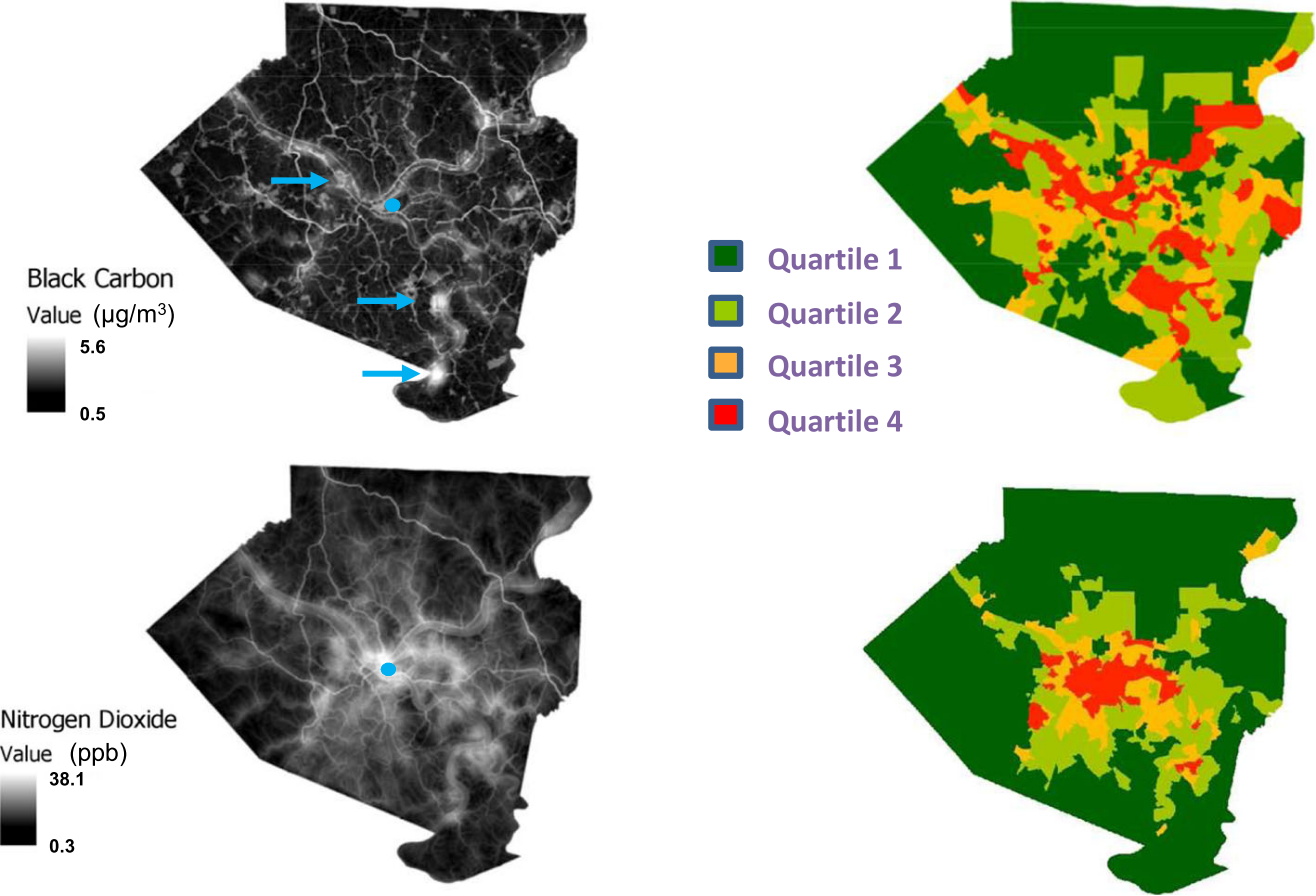
Exposure maps for black carbon and NO_2_ in Allegheny County during 2011–2012. Land use regression was applied to model pollutant concentrations as described in Methods. Left panels: represent the estimated distribution of the indicated pollutants as modeled over a grid containing cells sized at 50 m by 50 m. Pollutant concentrations are depicted over a grey scale with increasing concentrations represented by increased luminosity. The blue dots indicate the location of the Pittsburgh downtown business district, and blue arrows in the BC panel show locations of major industrial sources. An average exposure value for each census tract was calculated as the mean from all cells aggregated within that tract. Right panels show the distribution of census tract exposures divided into 4 quartiles of exposure magnitude where quartile 1 represents the least exposed and quartile 4 represents the highest

The LUR outputs are grid-cell averages for each cell, initially computed at 10 m resolution and then down-averaged. For example, each 50 m × 50 m cell in the final prediction raster can be divided into a grid of 36 nodes each separated by 10 m. We compute the circular buffers around each of those nodes to generate LUR predictions at each node, then compute the average across all nodes within each 50 m × 50 m grid cell. This average is what is shown in the high-resolution black and white maps in Fig. 1. The assigning of an exposure level to a given census tract was then taken as the arithmetic mean of all the grid cells contained within the boundaries of that tract.

### Demographic data and EJ designation

Identity of EJ-sensitive census tracts within Allegheny County were obtained from the Pennsylvania Department of Environmental Protection (PA-DEP) website for the Office of Environmental Justice [33]. At the time of this analysis the state defined these census tracts as containing greater than or equal to 30% of the resident population represented as non-white minority and/or greater than or equal to 20% of the population living below the Federal poverty limit. We also obtained a downloadable Excel file listing each EJ-designated tract and their delineation as meeting the race alone, poverty alone, or race + poverty criteria. Statistical methods used to derive these designations, however, were not detailed, but simply stated as “based on the most current census tract data from the U.S. Census Bureau and the federal guidelines for poverty”. This site was recently updated after the preparation of this manuscript and now contains updated EJ designations at the census block group level.

We further sought to validate these data independently in some way. Racial distribution estimates taken from the 2010 decennial census for each tract were used to determine % non-white minority in each tract. For poverty information, we were compelled to rely on the less accurate American Community Surveys (ACS). To assess the percent poverty in each tract we extracted the estimated total number of people in each tract along with the estimated people living in poverty from 2 different 5-year ACS estimates (2006–2010 and 2009–2013). This time period was chosen to approximate the time of the air monitoring campaign used to develop the exposure model. Coefficients of variations (CV%) were derived from the +/− error for each tract and expressed as a percent of the estimate. For details on methodology of validation see Supplemental Data. Average CV% for total population for all tracts were 6.5 and 6.4 and number in poverty were 35.0 and 31.9 for 2006–2010 ACS and 2009–2013, respectively. Inconsistencies between the PA-DEP listing and our independent validation were minor (see below) and we opted to use the original PA-DEP list for our risk-based comparisons.

### Integration of EJ and exposure determinations

In order to assess the relationship between EJ tract and pollutant exposure we first created an exposure continuum by rank ordering all 402 census tracts in Allegheny County from low to high based on the NO_2_ and BC exposure concentration. This distribution was then divided into 4 quartiles of exposure each containing 25% of the tracts. Specific EJ tracts were then highlighted along the exposure continuum and enumerated in each quartile. In order to determine whether EJ sensitive tracts were differentially distributed across the range of exposures we calculated the incidence ratio of EJ tracts occurring in each of the exposure quadrants (Q2, Q3, Q4) relative to the incidence on the lowest exposed quadrant (Q1). The calculation is similar to a typical relative risk calculation used in an exposure cohort study [34] with substitution of case and control definitions with EJ or non-EJ designation, respectively. Relative incidence of each Q_i_ (RI_i_) was calculated in Eq. 1:

1 $$ {\mathrm{RI}}_{\mathrm{i}}=\left[\left(\#{\mathrm{EJ}}_{\mathrm{i}}\right)/\left(\#{\mathrm{EJ}}_{\mathrm{i}}+\#\mathrm{non}-{\mathrm{EJ}}_{\mathrm{i}}\right)\right]/\left[\left(\#{\mathrm{EJ}}_1\right)/\left(\#{\mathrm{EJ}}_1+\#\mathrm{non}-{\mathrm{EJ}}_1\right)\right], $$

where 1 denotes lowest exposed quartile, Q1.

Statistically significant difference was taken as non-overlapping 95% confidence intervals.

### Risk-based modeling of CHD

For the purposes of developing a risk-based model to estimate disease burden from air pollution we chose a single well-defined health endpoint, coronary heart disease (CHD), for which we could readily obtain a measure for overall county-wide incidence and whose relationship to air pollution exposures had been previously quantified in multiple epidemiological health effect studies [11, 20, 35, 36]. Ischemic coronary heart disease (CHD) deaths were identified by cause of death on death certificates and hospitalizations were defined by ICD codes in hospital records (ICD9, 410–414 and 429.2 or ICD-10, 120–125) available to the county health department. According to the Allegheny County Health Department, crude coronary heart disease death rate in 2010 for Allegheny County, PA was 187.7 deaths per 100,000 people. Total hospitalizations for CHD over this same time throughout the county was 495 per 100,000 people. These overall risk values of CHD death (0.001877) and hospitalization (0.00495) obviously contain all components of risk beyond air pollution alone.

We next sought to parse out the components of risk that could be specifically attributed to air pollution. For this we relied on effect estimates provided in the study of Gan et al. [20], who conducted an epidemiological study examining the relationship of traffic-related pollutants to CHD hospitalization and mortality in Vancouver (BC, Canada). These authors developed specific quantitative effect estimates relating disease occurrence to long-term exposure to each specific pollutant after correcting for demographic variables (age, sex, comorbidity, and socio-economic status) and levels of co-pollutants. They estimated a 3 and 6% increase in CHD hospitalization and mortality, respectively for every 0.752 μg/m^3^ increment in BC exposure. For NO_2_, they calculated a 3% increase in CHD death for every 8.4 μg/m^3^ (4.47 ppb) increment in NO_2_. While a positive exposure-response relationship for hospitalization was evident after correction for demographic variables, it was not observed after correction for co-pollutants, so was not included for the present analysis.

To specifically address the air pollutant component of CHD risk in Allegheny County, we applied Gan et al.’s effect estimates [20] as follows. First, the overall county-wide risk of CHD death or hospitalization was arbitrarily assigned to the population-based midpoint exposure value of the county’s exposure continuum for BC or NO_2_ (i.e. the BC or NO_2_ exposure value of that census tract located at the point where 50% of the total population is at or above that value and 50% of the population is at or below that value). This relates the overall risk of disease to a midpoint county-wide standardized exposure value. The overall risk for each ascending and descending tract was then adjusted incrementally by the effect estimates of Gan et al. [20], applied to differences in exposure of that tract relative to midpoint. Therefore, the difference in risk within each tract relative to the population midpoint reference represents the augmentation or attenuation of risk arising from progressively higher and lower levels, respectively, of exposure relative to a countywide average. We then added the inverse of the negative risk in the lowest exposed tract as a constant to all other tracts. Thus, all resulting risk estimates are positive and expressed relative to the exposure in the lowest exposed tract. These risk values were then applied to the total census population of each tract to obtain a population-adjusted estimate of disease burden in each tract. A mathematical representation of the calculations described above is provided in the Supplemental Material (Equation S1). Risk values for each tract were summarized according to EJ or non-EJ designation, were found to not follow a normal distribution (D’Agostino & Pearson omnibus normality test), and then compared between each class using a non-parametric Mann-Whitney U-test.

## Results

Figure 1 shows the geographic distribution of NO_2_ and BC exposures across Allegheny County as estimated by the land-use regression modeling. At the highly resolved 50 × 50 m grid scale, discrete high levels of BC can be seen as white thread-like filaments extending throughout the county, which correspond to the major highways and transportation arteries. In addition, areas of high concentration of BC from major point sources, primarily coke processing and steel production, are also apparent (arrows). Distribution of NO_2_ appears more diffuse but still shows considerable heterogeneity throughout the county and again roughly tracks the location of major roadways and areas of high traffic density. When averaged at the level of census tracts, NO_2_ levels are highest in areas located toward the urban center of downtown Pittsburgh and tracts near major roadways and then progressively decrease as one moves toward the suburban margins. Similarly, the highest levels of BC are observed in areas characterized by either high traffic or the location of industrial point sources. Descriptive statistics describing the distribution of BC and NO_2_ census tract exposures including mean, median, and range for total and individual quartiles are provided in Tables S1, S2 (Supplementary Data). Quartiles were defined based on ranking along the exposure continuum for each pollutant. (e.g., Q1 contained the 25% of tracts with the lowest concentration).

**Fig. 2:**
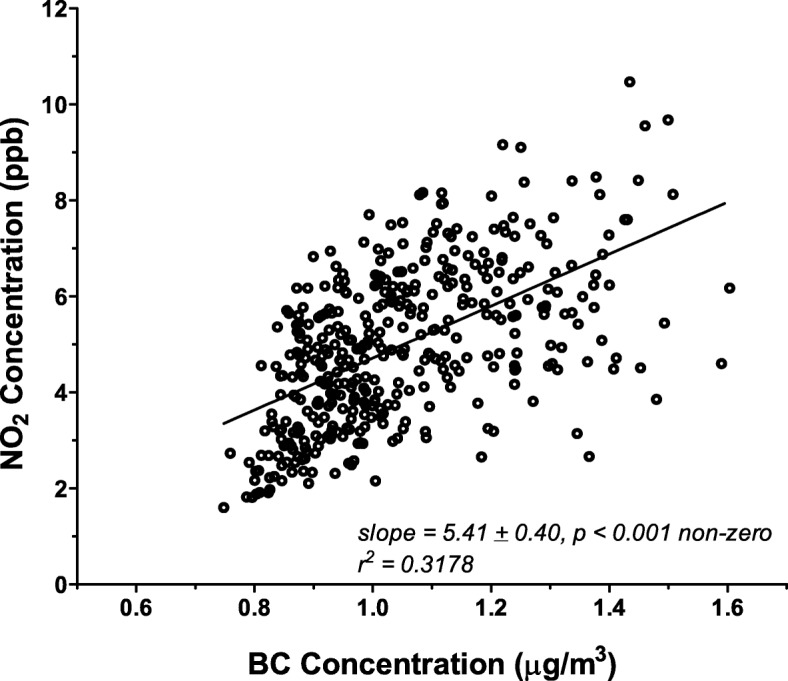
Correlation of BC and NO_2_ levels within Allegheny County census tracts. Exposure estimates for each pollutant were obtained for each census tract as described from the LUR models (Fig. 1). Each point represents a single tract plotted in terms of its modeled BC (μg/m^3^) and NO_2_ (ppb) value. Linear regression of the entire dataset indicates a significant positive correlation between each pollutant; however, considerable variability exists around the linear relationship (r^2^ = 0.3178)

Figure 2 shows the correlation between NO_2_ and BC exposure estimates between individual census tracts. As expected, there is a positive correlation between these two pollutants whereby higher concentrations of NO_2_ exposure are, in general, associated with higher concentrations of BC over the area. Significant variability, however, is apparent with numerous tracts falling far above or below the regression line. Linear regression of the relationship for co-dependence of these two pollutants on each other only explains approximately 30% of total variability in the distribution. Spearman’s rank correlation coefficient appropriate for non-Gaussian distributions was 0.58 indicative of only a moderate correlation between the two pollutants.

A comparison of EJ tracts designated by PA-DEP and their validation as described in Methods is shown in Tables S3 and S4 (Supplemental Data). PA-DEP listed 136 total EJ tracts, however, we found 134 based on the validation. The PA DEP classification accurately predicted EJ status based on race in every case. However, there were 13 tracts defined by PA-DEP as race alone that met our validation criteria of race and poverty. In addition, validation revealed 3 tracts defined only by race, but PA-DEP designated as race and poverty. Regardless, this difference does not materially change their inclusion as EJ tracts. Validation for poverty using combined 2006–2010 and 2009–2013 ACS indicated 5 tracts designated as EJ by PA-DEP based on poverty alone were slightly below the 20% poverty threshold and 3 previously unidentified tracts that met the poverty threshold that were not indicated as EJ by PA-DEP. Note that these last 3 tracts only barely exceed the 20% threshold. These discrepancies most likely arise from the inherent inaccuracies in the small-sample ACS estimates. Average CVs for the percent poverty estimates based on +/− errors in both total population and number below poverty were approximately 34% for both ACS surveys. It is not clear which versions of ACS the PA-DEP utilized or whether any thresholds were applied for CVs. While acknowledging these uncertainties we utilized only those EJ tracts listed as such by PA-DEP for further analysis.

**Fig. 3:**
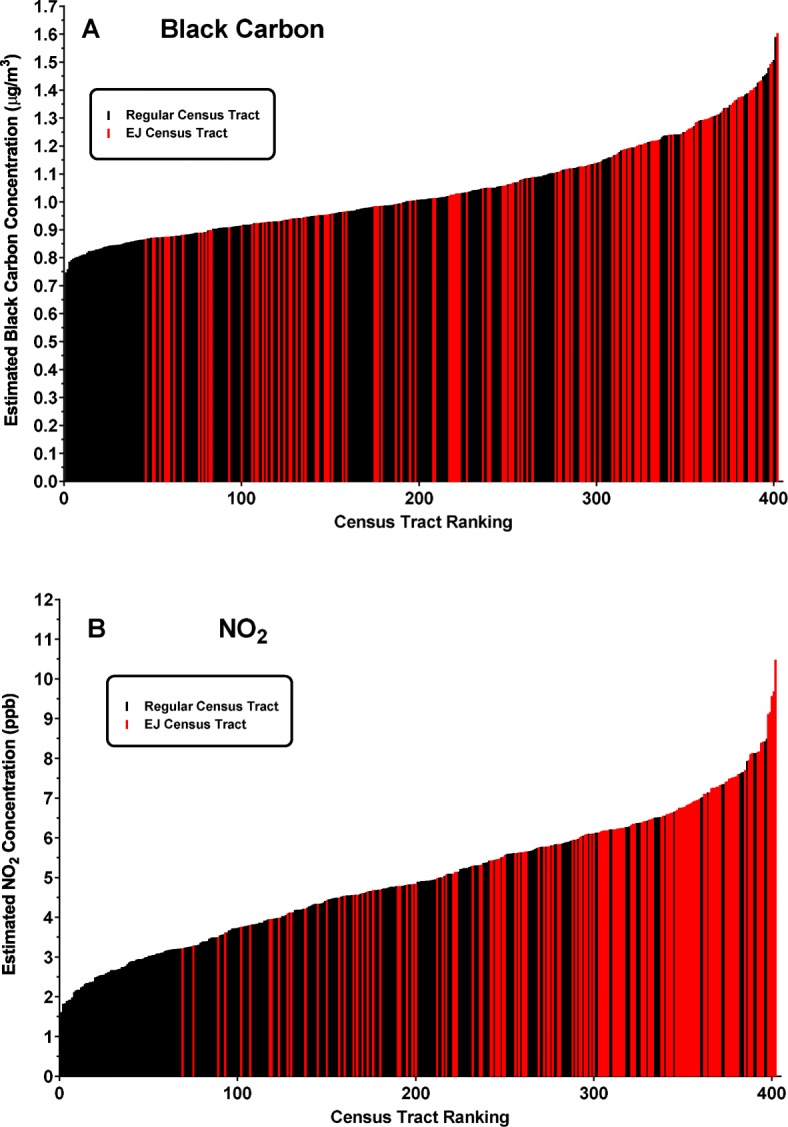
Distribution of EJ census tracts along the exposure continuum of black carbon (**a**) and nitrogen dioxide (**b**) in Allegheny County. Average long-term exposure estimates for each pollutant were derived for each census tract in the county following land-use regression as described in Methods. Based on the resulting exposure metric, tracts are ranked from lowest to highest exposure and those designated as EJ sensitive are displayed in red

Figure 3 shows the exposure continuums for each of the two pollutants. The 402 census tracts are ranked in increasing order based on average exposure estimates obtained from the LUR modeling. The red bars indicate those tracts that meet the criteria to be considered EJ areas. EJ tracts do not appear to be randomly distributed over the range of exposures. Very few EJ tracts are localized in the areas with the lowest exposure estimates, while a preponderance of EJ tracts are skewed towards areas of higher exposure. This is especially prominent for NO_2_.

We sought to develop a quantitative measure to describe EJ disparities. Each distribution was divided into quartiles (Q1 – Q4) of increasing exposure and the relative occurrence of EJ tracts in increasing quadrants of exposure (Q2 – Q4) relative to the lowest (Q1) was calculated. Table 1 shows that for BC, EJ tracts range from 2-fold more abundant in Q2 to almost 4-fold more abundant in Q4 compared to the lowest exposed quartile. This effect is even more pronounced for NO_2_, where Q2 contains 5.7 times, Q3 contains 8.7 times, and Q4 contains almost 20 times more EJ tracts relative to the first quartile. Thus, there appears to be a differential distribution of EJ tracts towards areas of higher exposure for both the BC and NO_2_ pollutants.

**Table 1:** Relative incidence of EJ Census Tracts in Allegheny County, PA increase as a function of pollutant exposure 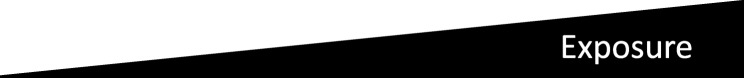

	Quartile 1	Quartile 2	Quartile 3	Quartile 4
Black Carbon	1	2.0 (1.2–3.6)	2.4 (1.4–4.1)	3.6 (2.2–5.9)
NO_2_	1	5.7 (2.0–15.9)	8.7 (3.2–23.5)	18.5 (7.1–48.7)

Data represent the fold-increases in the number of tracts meeting the criteria for EJ designation among the approximate 100 tracts represented in each quartile of increasing BC or NO_2_ exposure relative to the occurrence of EJ tracts in the lowest exposed quartile. Parentheses represent the 95% confidence interval for the determination

**Fig. 4:**
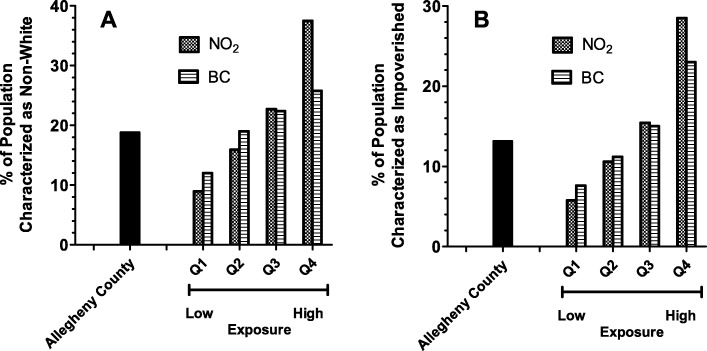
Percent of residents characterized as racial non-white minority (**a**) or impoverished (**b**) in various quartiles of pollutant exposure. Exposure continuums for each pollutant were divided into quartiles as described in Methods. Q1 represents the lowest exposed quartile and Q4 represents the highest. The total population as well as residents characterized as non-white or with incomes below the poverty line was determined for each census tract and then summed within each quartile. Racial minority and poverty were then expressed as a percent of the entire population within each quartile. The percentages of non-white and impoverished residents relative to the entire county population is shown for reference

Disparities of exposure by race and income are also noted when one considers the population of Allegheny County as a whole. Tables S5 and S6 (Supplemental Data) show total, racial (based on 2010 decennial census) and impoverished (based on average of 2010 and 2013 5-yr ACS estimates) population distributions for the entire county over the various quartiles of exposure for NO_2_ and BC. There are approximately 1.2 million people residing in Allegheny County of which 81.2% are white, 18.8% are non-white minority, and 13.1% live in poverty. There were progressively fewer total people living in the quartiles of higher exposure compared to lowest. For BC, 34% of the total population lives in Q1, compared to only 17.3% in Q4 (Table S5). Intermediate numbers reside in Q2 (27%) and Q3 (21.7%). This trend is mirrored by the white majority population of the county. In contrast, the non-white minority population is greater in the higher exposure quartiles, especially for NO_2_ (Table S6) with 16.5% in in Q1, 22.1% in Q2, 25.9% Q3, and 35.5% in Q4. Poverty shows a similar trend as race for both BC and NO_2_ with even greater gaps between the numbers of people below the poverty line residing in the highest quartiles of exposure compared to the lowest. Figure 4 considers the proportion of non-white minority (panel A) and impoverished (panel B) residents that compose each quartile of exposure for BC and NO_2_. In all cases, the composition of succeeding higher levels of exposure quartiles demonstrates that the percentages of non-white and economically disadvantaged people progressively increases 3–4 fold over the exposure range.

**Fig. 5:**
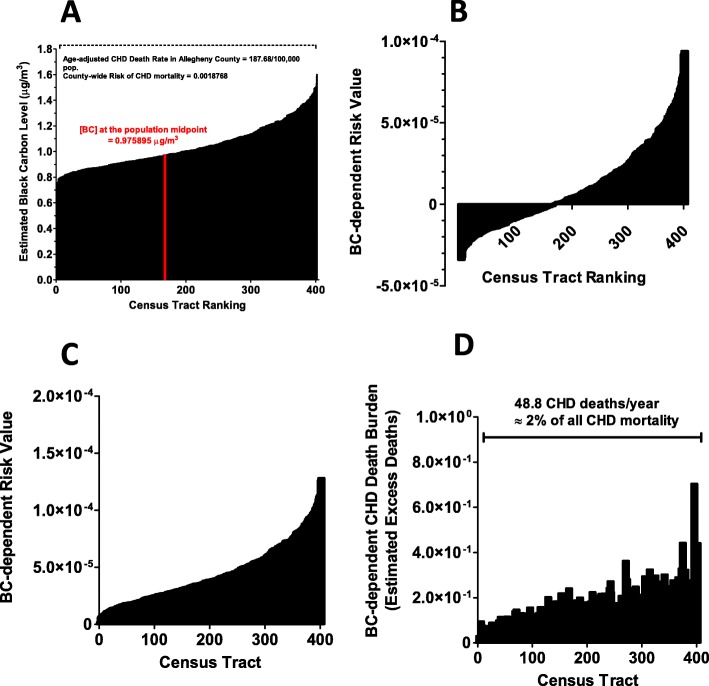
Steps used to develop a risk-based model of CHD burden from black carbon. **a** shows the exposure continuum for black carbon over all census tracts as described above. As a starting point, the overall age-adjusted average death rate for CHD for the entire county was arbitrarily assigned to the exposure metric for BC (0.975895 μg/m^3^) corresponding to the census tract that marks the population midpoint (ie., 50% of population lies below and 50% of population lies above this point). The overall risk of CHD death was then adjusted upward or downward based on the BC increment in each preceding and succeeding tract based on the BC effect estimate for CHD mortality reported by Gan et al. [20]. **b** shows the BC-dependent contributions to risk relative to the BC exposure present in the mid-population census tract. **c** expresses the BC-dependent risk value relative to the BC metric contained in the lowest exposed census tract. **d** shows the overall predicted BC-dependent CHD death burden in each tract by applying the risk values shown in panel C to the population of each census tract. When the disease burden is summed across all census tracts in the county, the model estimates a total of 48.8 CHD deaths arising from BC in excess of that present in the lowest exposed census tract, or 2% of the entire CHD deaths measured in the county

Next, we sought to develop a model in order to provide a risk-based estimate of disease burden attributed to air pollution within this area. Figure 5 shows a schematic flow-chart of the steps employed in this approach. The example depicted is for CHD mortality and BC exposure. First, we obtained the average death rate for CHD for all of Allegheny County (187.7/100,000 population) from 2010. We arbitrarily assigned this countywide average risk value (0.001877) to the population midpoint value of exposure as a surrogate for an average countywide exposure to each pollutant as described in Methods. Note that this represents the risk of CHD death from all causes and not just air pollution. The risk of CHD death in each tract is then adjusted upward or downward according the increase or decrease in pollutant exposure in each tract relative to this midpoint using the effect estimate of a 6% change in CHD mortality for every 0.752 μg/m^3^ increment change in BC described Gan et al. [20]. To estimate the relative pollutant-specific component within overall risk in each tract, the total risk value of the midpoint tract is subtracted from the total risk value in each of the other tracts. This leaves a residual component of risk that represents the estimated BC-specific effect relative to a county-wide average. In order to more appropriately visualize the detrimental effect of pollution over the entire county, we then asked what the pollutant-specific risks would be from pollutant levels in excess of the lowest exposed census tract. For this the BC-specific risks are then expressed relative to that in the lowest tract instead of the midpoint by adding the negative risk in the lowest tract as a constant to the BC-risk in all tracts (panel C). These final risk estimates are then applied to the population residing in each census tract to obtain an estimate of the CHD disease burden posed by pollutant exposure in each census tract (panel D). These can then be summed across the entire county or, specifically in EJ and non-EJ census tracts (as defined by the PA-DEP). Similar steps were employed for hospitalization rates using the appropriate effect estimates. Only CHD mortality was analyzed for NO_2_ since Gan et al. [20] failed to find a significant association between NO_2_ exposure and CHD hospitalization after correcting for co-pollutants.

**Fig. 6:**
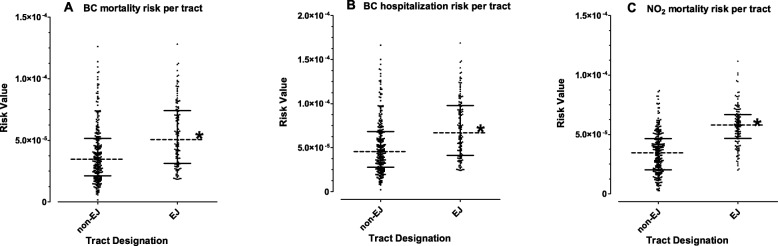
Comparison of pollutant-dependent CHD mortality and hospitalization risks between EJ and non-EJ census tracts in Allegheny County. The component of disease risk attributed to BC or NO_2_ was calculated for each census tract as described in Fig. 5. Data shows the distribution of all individual risk values compared between non-EJ (*n* = 265) and EJ (*n* = 136) tracts. Dotted lines represent the median and solid lines represent the interquartile range. Asterisks denote statistically significant difference between EJ and non-EJ tracts by Mann-Whitney U-test (*p* < 0.001)

Figure 6 shows the pollutant- and endpoint-specific risk values calculated for each individual census tract and compared between EJ and non-EJ tracts. In all cases, the average pollutant-dependent risk value was significantly higher for EJ tracts compared to non-EJ tracts. For BC, the risk for CHD mortality and hospitalization in approximately half of the EJ tracts was within the same range of only 25% of the highest exposed non-EJ tracts. For NO_2_, nearly 75% of EJ tracts are equivalent or greater than the highest exposed 25% of non-EJ tracts.

**Fig. 7:**
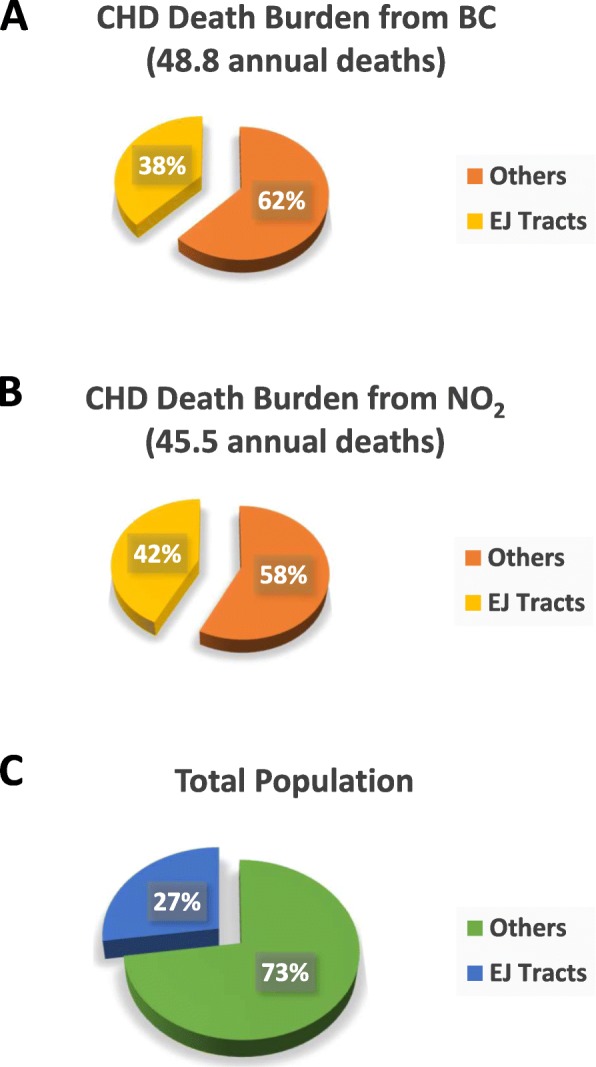
Risk-based estimate of CHD burden in EJ and non-EJ tracts in Allegheny County. Predicted annual cases of CHD mortality resulting from BC and NO_2_ exposure were calculated in each census tract in Allegheny County based on the pollutant exposure estimates, risk-based modelling, and total populations as described in Methods and Fig. 5. Overall, BC and NO_2_ were predicted to account for 49 and 46 CHD deaths, respectively, within the entire county. When divided into those estimated to occur in EJ tracts vs non-EJ regions, approximately 40% were represented in EJ tracts (38% for BC, **a**, 42% for NO_2_, **b**) despite the fact that the population of EJ tracts represents only 27% of the entire county population (**c**)

We estimated that, regarding the county as a whole, BC and NO_2_ exposures in excess of that present in the lowest exposed census tract were predicted to cause approximately 49 and 46 deaths, respectively from CHD annually. Together, these numbers represent approximately 4% of all countywide CHD deaths annually. Figure 6 shows the distribution of these predicted deaths between EJ and non-EJ tracts. With BC and NO_2_ it appeared that 38 and 42%, respectively, of the predicted pollutant-related CHD mortality burden was present within EJ sensitive areas (Fig. 7a, b). However, the population of EJ areas represents only 27% of the total population in the county (Fig. 7c). Black carbon was associated with approximately 64 predicted excess hospitalizations for CHD in the county per year (approximately 1% of the total), and again about 40% of those were represented within EJ tracts (data not shown).

## Discussion

Advocates of environmental justice have long appreciated the fact that environmental exposures are frequently increased amongst people of low socio-economic status and minority people of color, and by extension, they are likely to suffer greater burden of adverse health effects. Our data show that in Allegheny County, EJ sensitive areas are more numerous in areas of higher exposure and bear a disproportionate amount of risk of CHD from air pollution compared to non-EJ areas. These groups frequently are not adequately informed as to the nature of the environmental hazards present in their communities, lack an effective mechanism to voice concerns or advocate for change, and lack resources to move or otherwise improve their situation. While environmental health disparities have long been recognized and attempts made to address this at the policy level, inequities remain. Clark et al. recently compared NO_2_ exposures across the entire US for the years 2000 and 2010 based on race-ethnicity and socioeconomic status [25]. While significant reductions on NO_2_ were observed across all areas and all groups over this time, marked disparities by race and income remained. Overall, the non-white to white disparity declined from 5.0 ppb in 2000 to 2.9 in 2010; however, on a relative basis, non-whites remained 37% more-exposed than whites in 2010 compared to 40% in 2000. In fact, PM_2.5_ and NO_2_ exposure inequities may have actually broadened in some areas [37]. Thus, policies typified by the Clean Air Act to reduce air pollution over the entire population do little to reduce social and economic disparities.

Clark et al. found stronger disparities based on race and ethnicity than on income [25]. Since most of the EJ tracts in Allegheny County (49%) are defined by both parameters of 30% minority and 20% poverty, it was difficult for us to address the relative role of each alone. We did, however, compare all PA-DEP defined EJ tracts that included the race factor (regardless of income) to those defined by income (regardless of race) and found stronger associations between race and higher exposure than we did for income (data not shown).

Understanding the sources of these exposures will be important to develop and implement strategies targeted towards reducing exposures amongst EJ communities and normalize the disparities throughout the county. NO_2_ is most often considered a traffic-related pollutant, while BC contains significant contributions from both mobile and point sources. EJ communities are, in general, subjected to greater traffic-related pollution compared to other areas. The early transportation policies of the 1970’s were characterized by rapid expansion of high-density freeways and thoroughfares that transect or encircle major urban centers [38, 39]. Such construction was geared to meet the demands of urban sprawl with delivery of goods and services to and from large urban centers and long-distance commuting of workers, but gave rise to significant increases in diesel truck traffic as well as high-number of single-occupancy vehicles. Such policies frequently failed to consider the impact on communities closest to their construction, which are often composed of those groups least able to afford the suburban migration predominated by affluent whites. Traffic–related air pollutants can remain elevated up to 500–1000 m from the roadway [40, 41]. Rowangould found that racial minorities and low-income households were more likely to live near high traffic roadways than the general population [42].

The Q4 areas for both BC and NO_2_ shown in Fig. 1, however, are a mix of both high traffic and industrially-impacted areas. Recently, Mikati et al. showed disparities by race and poverty status regarding proximity to major industrial point sources of PM_2.5_ [43]. Thus, it is likely that industrial emissions also contribute significantly to the disparities in exposure seen here. In addition, Allegheny County geography is predominated by multiple large river valleys in which major transportation arteries (including rail and barge traffic), major industrial point sources, and socio-economically disadvantaged neighborhoods are co-localized in low-lying regions prone to pollution trapping.

Although there is some co-localization of the two pollutants considered here, for the overall estimation of CHD disease burden we considered NO_2_ and BC as independent factors whose combined effects were likely additive. While direct effects of PM_2.5_ on a variety of health endpoints has been well established, it appears that NO_2_ also has effects independent of other co-pollutants [44]. We applied risk estimates for each that had been previously corrected for co-pollutant contributions [20]. Moreover, in our exposure distributions we observed considerable departure in the co-dependence of each pollutant (Fig. 2) with numerous census tracts showing an elevation of one but not the other pollutant leaving nearly ¾ of the variation in each unexplained by co-dependence of the other pollutant. Thus, the overall burden of disease needs to consider the totality of independent contributions from multiple pollutants.

Our model also assumes a linear dose-effect relationship over the range of pollutant concentrations expressed here, although there may be significant departure from linearity at extremes of exposures experienced in other parts of the world [45]. Of note is the observation that a positive association between adverse effects and pollutant concentrations below ambient standard has been demonstrated [46], especially for the elderly, and scientific consensus holds that no threshold for such adverse effects has been demonstrated. Predicting the effect estimate at very low pollutant levels may be controversial; however, we modeled our disease burden calculations relative to the exposure in the lowest census tract, and not to the elimination of pollutant entirely from the ambient environment.

Lacking any more definitive quantitative dose-response function applicable to long-range exposures over this relatively narrow exposure range, we feel application of a linear model is a reasonable initial approach. First, the rise in health of effects of BC-dependent hospitalization and mortality and NO_2_-dependent mortality appear linear over the quintiles of exposure measured in Gan et al. [20]. Moreover, while studies measuring PM_2.5_ [47, 48] show complex biphasic dose-responses characterized by steeper linear dose-response at low doses and attenuation of effect at higher dose extremes, the relative narrow range of BC concentrations encountered in both Gan et al. [20] and our study would likely fall in the steeper portions of the PM_2.5_ curves that approximate linearity. Bai et al. [49] present a dose-response for NO_2_ and myocardial infarction that approximates linearity between 2 and 10 ppb and then decreases its slope as doses exceed 10 pbb. If the effect estimate of Gan et al. [20], is over-represented from the higher part of this curve, that could lead to an underestimation of NO_2_ burden estimated here, as Allegheny County NO_2_ concentrations actually would have greater representation from the steeper part of that curve than those of Gan et al. [20]. The fact that the BC had a greater impact on CHD mortality (2% of total) compared to CHD hospitalization (1% of total) within the context of greater number overall hospitalizations compared to deaths was somewhat surprising. This arises from the stronger effect estimates for mortality compared to hospitalization derived by Gan et al. [20]. In fact, NO_2_ failed to have a significant effect on hospitalization in that study despite having a positive impact on mortality. One explanation may be that the untoward cardio-vascular effects of air pollution may be more significant in older individuals or those with severe pre-existing disease where triggering an adverse event by pollutant exposure may have a disproportionate lethal effect compared to those with early, often subclinical, stages of disease. Future health effect studies should take age, pre-existing conditions, and disease stage and severity into consideration when possible to address how these factors may modify the effect of air pollution. In addition, we used baseline CHD mortality and hospitalization rates representative of the entire county population. It might be that additional risk factors in racial minorities and the poor may inflate the overall rate of disease in these groups and lead to an underestimation of EJ disparity in disease burden reported here.

The overall precision of calculating disease burdens also depends on validity of LUR models. First, the exposure estimates reflect projections of long-term average exposure concentrations, hence they ignore short-term fluctuations in concentrations that may also play a role in initiating untoward cardiovascular events [18]. Our LUR model incorporating mobile source plume analysis and mobile sampling may improve model application to traffic-related pollutants, but may underestimate the contribution from fixed point sources [50]. Hence, the estimated overall effect of BC may under-represent those adverse effects arising from industrial BC emissions from such facilities as coke and steel plants.

Some uncertainty in our risk estimate also arises with the use of effect estimates derived from a single study. Surveying several meta-analyses [28, 51] yielded no additional studies providing suitable effect estimates corresponding to long-term exposure specifically for BC and the CHD endpoint. More information was available from a similar survey of NO_2_ studies [27]. The mean hazard ratio of 15 studies examining cardiovascular mortality and NO_2_ collected in the meta-analyses by Atkinson et al. [27] was 1.03 / 10 μg/m^3^ and fell within a 95% confidence interval of 1.02 and 1.05. Applying these lower and upper bounds for NO_2_-dependent CHD mortality to our risk model predicts a range of from 25 to 63 excess deaths per year compared to our single calculation of 46 deaths based on the single effect estimate of Gan et al. [20].

Another limitation is that the effect estimates were drawn from an analysis [20] not conducted within the same geographic area as Allegheny County. Although both Vancouver and Pittsburgh are urban centers of moderate size, differences in pollution sources and other factors may mean that actual concentration-response estimates may not be identical in each locale. In comparing the estimated pollutant exposures between our study and those of Gan et al. [20] we noted similar mean values for BC of 1.19 and 1.05 μg/m^3^ for Vancouver and Allegheny County, respectively, although the range of values in Vancouver is greater as evidenced by an IQR of 0.75 μg/m^3^ compared to only 0.22 μg/m^3^ in our study. Greater differences in NO_2_ were observed between both studies. Mean NO_2_ in Allegheny county was 4.96 ppb but over 3-fold higher (17.7 ppb) in Vancouver. Again greater variation is seen in Vancouver with a 2-fold greater IQR (4.47 ppb) compared to Allegheny County (2.47 ppb). The reasons for this are beyond the scope of this discussion but may reflect differences in modeling parameters (residential points vs. census tracts), different sampling periods (late 1990s vs. 2010), and differences in population density, study area size, and pollution sources.

Lastly, it should be pointed out that variables derived from ACS data can contain significant uncertainty based on rather large CV% in some areas. We chose to retain the PA-DEP designations since they are the most applicable to policy decisions at the regulatory level, however, the statistical methods applied in their use of ACS data are not available. We have shown that the choice of various ACS datasets can influence some EJ determinations especially regarding income. This variance however likely makes little difference in our results here. First, all EJ tracts characterized by race and poverty, would still meet the EJ definition by race regardless of what actual percent poverty was. By comparing the PA-DEP listing to our ACS analysis we find only a net under-counting of 2 tracts. This small percentage of total would have little impact in our estimate of EJ incidence within exposure quartiles or overall burden of disease within the population. Nonetheless, depending on the purpose of the research and for conducting future sensitivity analyses researchers may consider applying various arbitrary CV% thresholds for flagging potentially unreliable data [52, 53]. However, it should be pointed that higher CV%s are frequently are encountered in low income, minority, and urban neighborhoods [54] and a systematic exclusion of these data could itself bias the outcome.

## Conclusion

Our study provides a useful tool by which communities can assess the potential impact of air pollution once provided with an adequate representation of long-term pollutant exposure on a highly-resolved spatial scale. Of course the ability to apply this model depends on access to a suitable air pollution exposure model with high spatial resolution for the area of concern. Publically-available datasets for certain pollutants are becoming increasingly available. Advances in satellite imaging offer increasing geospatial resolution for measuring certain pollutants over large areas. Widespread deployment of low-cost air sensor networks in communities could be used in conjunction with land use regression modeling to construct similar pollutant maps to ours with minimal technical expertise once criteria of instrument reliability and sensitivity are established. Use of a risk-based model allows a timely estimate of the health burden as well as temporal tracking of the potential impacts of mitigation strategies when before and after exposure estimates are available. It will, however, be important to validate the risk-based model using historical or prospective disease data in future studies. Also, a quantitative measure of EJ disparities as employed here is useful to be able to compare different communities, as well as track progress in various corrective public policies. It is important to state that our risk-based estimates presented here refer only to a single disease, namely CHD. Since these same air pollutants have been associated with numerous other serious diseases, many with lethal outcomes, it should be noted that the overall impact of air pollution in this study area is likely much greater than that suggested by the limited analysis here. Future work can now be undertaken to estimate the impact of air pollution on other disease outcomes using our risk-based model and exposure estimates in conjunction with quantitative effect estimates for other diseases available in existing or forthcoming health outcome studies.

## Supplementary information

**Additional file 1.**

## Data Availability

Population and demographic data for census tracts contained in Allegheny County are publically available and accessible through the U.S. Census Bureau (Census.gov). The datasets used for the LUR modeling of pollutant exposure are available from the corresponding author on reasonable request.

## Abbreviations

BC: Black Carbon
CHD: Coronary Heart Disease
EJ: Environmental Justice
LUR: Land Use Regression
NO_2_: Nitrogen Dioxide
PA DEP: Pennsylvania Department of Environmental Protection
PA: Pennsylvania
PM: Particulate Matter
US EPA: United States Environmental Protection Agency
