# Supplementary material for: A risk-based model to assess environmental justice and coronary heart disease burden from traffic-related air pollutants
Source: Environ Health. 2020 Mar 16;19:34. doi: 10.1186/s12940-020-00584-z (PMC7075037; doi:10.1186/s12940-020-00584-z)
Supplement: Supplementary file 1 — Additional file 1. [file 12940_2020_584_MOESM1_ESM.pdf]

SUPPLEMENTAL MATERIAL

A RISK-BASED MODEL TO ASSESS ENVIRONMENTAL JUSTICE AND CORONARY HEART DISEASE BURDEN FROM TRAFFIC-RELATED AIR POLLUTANTS

James P. Fabisiak[1]

Erica M. Jackson[1]

LuAnn L. Brink[2]

Albert A. Presto[3]

[1]Center for Healthy Environments & Communities[1], Department of Environmental & Occupational Health, University of Pittsburgh Graduate School of Public Health, Pittsburgh, PA

[2]Allegheny County Health Department, Pittsburgh, PA

[3]Center for Atmospheric Particle Studies, Department of Mechanical Engineering, Carnegie Mellon University, Pittsburgh, PA

Equation S1. Mathematical representation of the black carbon (BC) component of CHD mortality risk in Allegheny County census tracts.

$$\textbf{BC risk}_i = (((\text{[BC]}_i - \text{[BC]}_{midpoint})/0.752) \times 0.06) \times \text{CHD risk}_{county} + \text{BC risk}_{lowest}$$

Where,

BC $risk_i$ = Black carbon component of CHD risk in tract i

$[BC]_i$ = Black carbon concentration in tract i

$[BC]_{midpoint}$) = Black carbon concentration at the population midpoint tract

CHD $risk_{county}$ = Overall county wide CHD death rate assigned to the population midpoint tract. For hospitalizations, this term is replaced with overall county CHD hospitalization rate.

0.752 and 0.06 are derived from the effect estimates of Gan et al. (6% in CHD death for every 0.752 $\mu g/m^3$ of BC). Corresponding values for NO_2 and CHD hospitalization can also be found in Gan et al. (Gan et al. 2011)

BC $risk_i$ provides a unitless risk estimate for each census $tract_i$ that can then be applied to the population of that tract to give a predicted value for the number of BC-dependent CHD deaths in that $tract_i$. Predicted number of deaths are then summed across all census tracts in the county or segregated by EJ and non-EJ designated tracts.

Table S1. Descriptive statistics for the distribution of BC exposure estimates (μg/m^3) across census tracts in Allegheny County, PA.

	Total	Q1	Q2	Q3	Q4
**Mean**	1.046	0.863	0.963	1.071	1.289
**Median**	1.009	0.875	0.959	1.108	1.263
**Range**	0.748 - 1.604	0.748 - 0.915	0.918 - 1.009	1.009 - 1.142	1.146 - 1.604
**IQR**	0.224	-	-	-	-

Table S2. Descriptive statistics for the distribution of NO_2 exposure estimates (ppb) across census tracts in Allegheny County, PA.

	Total	Q1	Q2	Q3	Q4
**Mean**	4.965	2.922	4.357	5.514	7.069
**Median**	4.889	3.025	4.448	5.593	6.824
**Range**	1.605 - 0.471	1.605 - 3.726	3.737 - 4.888	4.890 - 6.117	6.131 - 10.471
**IQR**	2.377	-	-	-	-

Tables S3, S4: Comparison of EJ tracts in Allegheny County as defined by PA-DEP and independent validation using 2010 decennial census and 2006 – 2010 and 2009 – 2013 5-yr ACS as described in METHODS. See methodology for validation immediately following S3 and S4.

S3: Comparison of the number of census tracts meeting the EJ thresholds of poverty, race, or both.

Designation	> 30% non-white	> 20% in poverty	> 30% non-white<br>> 20% in poverty	Total
**PA-DEP**	33	36	67	136
**Using ACS poverty from 2010 + 2013**	23	34	77	134

S4: Identity of tracts with discrepancies between EJ status as delineated by PA-DEP and independent validation.

Census Tract	PA-DEP designation	With 2010/2013 ACS Designation	Calculated % Poverty with 2010/2013 ACS (Error %)
709	Race only	Both Race and Poverty	**23.1% (18.9%)**
2107	Race only	Both Race and Poverty	**25.1% (27.8%)**
4868	Race only	Both Race and Poverty	**22.9% (24.2%)**
5616	Race only	Both Race and Poverty	**32.9% (29.9%)**
5617	Race only	Both Race and Poverty	**43.0% (30.5%)**
5619	Race only	Both Race and Poverty	**25.3% (31.8%)**
5620	Race only	Both Race and Poverty	**38.4% (22.8%)**
5623	Race only	Both Race and Poverty	**27.5% (22.2%)**
5625	Race only	Both Race and Poverty	**33.7% (22.3%)**
5626	Race only	Both Race and Poverty	**22.2% (28.1%)**
5627	Race only	Both Race and Poverty	**35.3% (20.1%)**
5632	Race only	Both Race and Poverty	**31.1% (20.9%)**
9818	Race only	Both Race and Poverty	**87.0% (73.1%)**
1017	Both Race and Poverty	Race only	**16.9% (34.9%)**
1405	Both Race and Poverty	Race only	**15.8% (36.2%)**
5614	Both Race and Poverty	Race only	**19.7% (23.7%)**
2406	Poverty only	Non-EJ	**16.9% (29.6%)**
4135	Poverty only	Non-EJ	**12.1% (40.2%)**
4324	Poverty only	Non-EJ	**15.5% (25.8%)**
4706	Poverty only	Non-EJ	**17.8% (25.4%)**
5120	Poverty only	Non-EJ	**16.5% (34.1%)**
4507	Non-EJ	Poverty only	**20.8% (33.0%)**
4689	Non-EJ	Poverty only	**20.7% (22.1%)**
4882	Non-EJ	Poverty only	**20.7% (21.7%)**

Methodology for validating EJ tract characterization. PA-DEP EJ designations were obtained as described in METHODS. Racial data for all Allegheny County census tracts was obtained from the 2010 Decennial Census. Percent non-white minority living in each tract was calculated as [(total population of $tract_i$ / total non-white population in $tract_i$) X 100]. This calculated percentage was then compared to the PA-DEP criteria of > 30% and found to match completely with PA-DEP delineations in all EJ tracts. Numbers of people in poverty were estimated in Allegheny County census tracts from 2 separate versions of the 5-yr American Community Survey (ACS) (2006 – 2010 & 2009 – 2013). For each version of the ACS we calculated as follows:

% people in poverty $tract_i$ = (# in poverty $tract_1$ / # total population $tract_i$) X 100

SE # total population in $tract_i$ = MOE # total population $tract_1$ /1.645 (see ACS documentation in references)

CV for # total population $tract_i$ = SE # total population $tract_i$ / # total population $tract_i$

SE for # in poverty $tract_i$ = MOE for # in poverty $tract_i$ /1.645

CV for # in poverty $tract_i$ = SE for # in poverty $tract_i$ / # in poverty $tract_i$

CV for % people in poverty $tract_i$ = $\sqrt{(\text{CV \# in poverty tract})^2 + (\text{CV \# total population in tract})^2}$

CVs can be converted to CV% by multiplying by 100

SE = standard error of the estimate, MOE = margin of error of the estimate, CV = coefficient of variation for the estimate.

A final result combining both ACS versions for comparing to PA-DEP designations was obtained by averaging the calculated % poverty and CV% for % poverty from the 2 different ACS.

Table S5: Racial and economic demographics by quartiles of BC exposure in Allegheny County, PA.

	County	1st Quartile	2nd Quartile	3rd Quartile	4th Quartile
**Total Population (% of county)**	1,222,708	384,055 (31.4%)[1]	330,526 (27%)[1]	277,811 (22.7%)[1]	230,316 (18.8%)[1]
**White Population (% of county)**	992,824 (81.2%)[1]	337,958 (34.0%)[2]	267,904 (27.0%)[2]	215,559 (21.7%)[2]	171,403 (17.3%)[2]
**Non-White Population (% of county)**	229,884 (18.8%)[1]	46,097 (20.1%)[2]	62,622 (27.2%)[2]	62,252 (27.1%)[2]	58,913 (25.6%)[2]
**People Below Poverty (% of county)**	160,678 (13.1%)[1]	29,003 (18.1%)[2]	37,061 (23.1%)[2]	41,614 (25.9%)[2]	53,000 (33.0%)[2]

[1]Denotes percent of total population in Allegheny County.
[2]Denotes percent of total for that sub-group in Allegheny County

Table S6. Racial and economic demographics by quartiles of NO_2 exposure in Allegheny County, PA.

	County	1st Quartile	2nd Quartile	3rd Quartile	4th Quartile
**Total Population (% of county)**	1,222,708	423,883 (34.7%)[1]	319,215 (26.1%)[1]	261,720 (21.4%)[1]	217,890 (17.8%)[1]
**White Population (% of county**	992,824 (81.2%)[1]	386,007 (38.9%)[2]	268,377 (27.0%)[2]	199,503 (20.1%)[2]	136,189 (13.7%)[2]
**Non-White Population (% of county)**	229,884 (18.8%)[1]	37,876 (16.5%)[2]	50,838 (22.1%)[2]	59,469 (25.9%)[2]	81,701 (35.5%)[2]
**People Below Poverty (% of county)**	160,678 (13.1%)[1]	24,429 (15.2%)[2]	33,759 (21.0%)[2]	40,378 (25.1%)[2]	61,112 (38.7%)[2]

[1]Denotes percent of total population in Allegheny County.
[2]Denotes percent of total for that sub-group in Allegheny County

REFERENCES

Gan WQ, Koehoorn M, Davies HW, Demers PA, Tamburic L, Brauer M. 2011. Long-term exposure to traffic-related air pollution and the risk of coronary heart disease hospitalization and mortality. Environ Health Perspect 119:501-507.

U.S. Census Bureau. 2018. Understanding and Using American Community Survey Data. What all Data Users Need to Know. U.S. Government Printing Office, Washington, DC. Available at, https://www.census.gov/programs-surveys/acs/guidance/handbooks/general.html.
